# Genetic variants in ADAM33 are associated with airway inflammation and lung function in COPD

**DOI:** 10.1186/1471-2466-14-173

**Published:** 2014-11-04

**Authors:** Xinyan Wang, Wan Li, Kun Huang, Xiaowen Kang, Zhaoguo Li, Chengcheng Yang, Xiaomei Wu, Lina Chen

**Affiliations:** Department of Respiratory, the Second Affiliated Hospital of Harbin Medical University, Harbin, 150081 China; College of Bioinformatics Science and Technology, Harbin Medical University, Harbin, 150081 China

**Keywords:** Chronic obstructive pulmonary disease, SNP, ADAM33, Haplotype, Pulmonary function, Airway inflammatory process

## Abstract

**Background:**

Genetic factors play a role in the development and severity of chronic obstructive pulmonary disease (COPD). The pathogenesis of COPD is a multifactorial process including an inflammatory cell profile. Recent studies revealed that single nucleotide polymorphisms (SNPs) within ADAM33 increased the susceptibility to COPD through changing the airway inflammatory process and lung function.

**Methods:**

In this paper, we investigated associations of four polymorphisms (T1, T2, S2 and Q-1) of ADAM33 as well as their haplotypes with pulmonary function and airway inflammatory process in an East Asian population of patients with COPD.

**Results:**

We found that T1, T2 and Q-1 were significantly associated with the changes of pulmonary function and components of cells in sputum of COPD, and T1 and Q-1 were significantly associated with cytokines and mediators of inflammation in airway of COPD in recessive models. 10 haplotypes were significantly associated with transfer factor of the lung for carbon monoxide in the disease state, 4 haplotypes were significantly associated with forced expiratory volume in one second, and other haplotypes were associated with airway inflammation.

**Conclusions:**

We confirmed for the first time that ADAM33 was involved in the pathogenesis of COPD by affecting airway inflammation and immune response in an East Asian population. Our results made the genetic background of COPD, a common and disabling disease, more apparent, which would supply genetic support for the study of the mechanism, classification and treatment for this disease.

**Electronic supplementary material:**

The online version of this article (doi:10.1186/1471-2466-14-173) contains supplementary material, which is available to authorized users.

## Background

Chronic obstructive pulmonary disease (COPD) is defined as a disease state characterized by poorly reversible and progressive airflow limitation that is usually associated with an abnormal inflammatory response of the lung [1, 2]. Nearly 90% of COPD is caused by long term cigarette smoking, however, only 25% of chronic tobacco smokers develop COPD [3]. Additionally, COPD tends to occur more frequently in smokers with a family history of obstructive airways disorders including asthma and COPD. All of these suggest that, besides smoking, there are other underlying genetic factors in the development of COPD. Variants of some genes have been investigated and identified to have close associations with COPD, such as TLR-9 [4], HHIP [5], IREB2 and CHRNA3/5 [6].

The pathophysiology of COPD is a multifactorial process with an complex inflammatory cell profile including eosinophils [7], macrophages [8], neutrophils [9], and lymphocytes [10]. The levels of some cytokines, such as interleukin(IL)-8 [11], interleukin(IL)-6 [12], tumor necrosis factor alpha (TNF-A) [13], and vascular endothelial growth factor (VEGF) [14] are increased in stable COPD patients, suggesting their key-roles in the pathogenesis of COPD. Therefore, COPD develops as a result of multiple steps involving the inflammatory cells and mediators, in which local inflammation in the lungs is especially important due to affecting airway remodeling and parenchymal destruction.

A disintegrin and metalloprotease 33 (ADAM33), a member of the ADAM (a disintegrin and metalloprotease) family, has been identified as an asthma susceptible gene [15]. This finding has been further improved in many populations, such as Indian and Chinese asthma populations [16, 17]. ADAM proteins are involved in cell adhesion, cell fusion, cell signaling, and proteolysis [18–20]. The latter can be illustrated by the capacity to shed cytokines, growth factors, or their receptors from the cell surface, and the remodeling of extracellular matrix components. Garlisi and colleagues demonstrated that ADAM33 is an active proteinase that is able to cleave α2-macroglobulin [21], an important member of pulmonary defense system. These results suggested that ADAM33 is involved in the pathogenesis of airway obstruction with affecting tissue remodeling, a physiological process intricately related to airway inflammation [22]. Additional studies have demonstrated that single nucleotide polymorphisms (SNPs) within ADAM33 were associated with accelerated decline of lung function in the general population and in asthma patients [23, 24]. Recent studies further revealed that SNPs within ADAM33 conferred susceptibility to COPD in the general population through affecting the airway inflammatory process and changing the lung function in COPD [25–28]. Our previous study has further confirmed the association between ADAM33 gene polymorphisms and COPD in the Northeastern Chinese Han population [29]. The aim of this study is to investigate the associations of SNPs in ADAM33 with the severity of the pulmonary function and airway inflammation in patients with COPD.

## Methods

312 patients with stable COPD were recruited for this study. The criteria of the recruitment have been described in detail previously [29], and are shown in Table 1. In brief, all patients had irreversible airflow limitation and chronic respiratory symptoms, and were current or ex-smokers with at least 20 pack-years of smoking. Patients did not use a course of inhaled or oral corticosteroids within 3 months, or maintenance treatment with these drugs within 6 months. None of the patients had a history of asthma. Patients with acute exacerbations two months preceding study assessment were also excluded. Disease severity was classified according to the criteria of Global Initiative for Chronic Obstructive Lung Disease (GOLD) [1]. Approval from the Ethics Committee of Harbin Medical University was obtained before initiating the study. COPD patients were informed of the study protocol and provided written consent.

**Table 1:** **The clinical information of patients recruited**

	CASE ^*^	CONTROL
Number of patients	312	319
Age (years)	60.5 (7.8)	61.5 (8.1)
Male: Female	186:126	192:127
Pack years of smoking^†^	35.46 (15.9)	32.54 (12.7)
FEV1 (% predicted)^‡^	52.5 (8.6)	91.5 (9.6)
FEV1/FVC (%)^§^	47.5 (7.6)	90.5 (11.6)
Postbd FEV1 (% predicted)^||^	58.5 (9.6)	93.5 (10.5)
Postbd FEV1/FVC (%)	49.2 (8.5)	91.5 (11.8)
TLCO (% predicted)**	63.09 (18.1)	93.21 (2.9)
GOLD status		
Stage I (mild)	52	-
Stage II (moderate)	140	-
Stage III (severe)	98	-
Stage IV (very severe)	22	-

^*^Data are means and standard deviations (in parentheses).

^†^Pack year: (packs per day) × (years smoked).

^‡^FEV1: forced expiratory volume in first second.

^§^FVC: forced vital capacity.

^||^Postbd: post bronchodilator.

^**^TLCO: transfer factor of the lung for carbon monoxide.

DNA was extracted from peripheral blood of both COPD patients and control samples, and genotyping was performed as described previously [29] using polymerase chain reaction-restriction fragment length polymorphism (PCR-RFLP) analysis. Based on our previous study [29], the four SNPs in ADAM33 have been genotyped: Q-1(C/T), S2 (G/C), T1 (Met-Thr), T2 (Pro-Ser) and the primers used are listed in Table 2.

**Table 2:** **The location of investigated ADAM33 SNPs and primer sequence**

Chromosome Position	Reference SNP ID	SNP Name	Alleles	Primer sequences
3590205	2280090	T2	A/G	F: 5′-TTCTCAGGGTCTGGGAGAAA-3′
				R: 5′-GCCAACCTCCTGGACTCTTA-3′
3590234	2280091	T1	A/G	F: 5′-ACTCAAGGTGACTGGGTGCT-3′
				R: 5′-GAGGGCATGAGGCTCACTTG-3′
3591742	528557	S2	C/G	F: 5′-AGAGCTCTGAGGAGGGGAAC-3′
				R: 5′-TGTGCAGGCTGAAAGTATGC-3′
3592207	612709	Q-1	A/G	F: 5′-GGATTCAAACGGCAAGGAG-3′
				R: 5′-GTTCACCTAGATGGCCAGGA-3′

### Sputum induction and processing

Sputum samples were obtained in the stable clinical state of COPD patients, at least two months after any exacerbation. Following the established standards for inducing sputum [30], patients inhaled 200 ug of salbutamol and afterwards 3% saline from an ultrasonic nebulizer for three 10 min periods, unless a 20% drop in forced expiratory volume in one second (FEV1) occurred. If this occurred, consecutive sputum inductions were performed using 0.9% saline. Samples produced after the three inhalation periods were processed separately and pooled values were derived by weighted averaging [31].

All adequate plugs of sputum were selected. After incubation with Sputolysin TM, Giemsa-stained cytospins were prepared. Non-squamous cells were counted by two independent observers, and the average of the values was taken for analysis. The levels of IL-6, IL-8, TNF-a and VEGF in sputum supernatants were determined in adequately diluted samples by ELISA (U.S. TPI kit).

### Lung function measurement

Pulmonary function testing of both COPD patients and control samples was evaluated on a Jaeger Transfer screen II (Erich Jaeger GmbH, Würzburg, Germany) and included: total lung volume; FEV1; vital capacity (VC); forced vital capacity (FVC); peak expiratory flow (PEF); and transfer factor of the lung for carbon monoxide (TLCO). Evaluation of the pulmonary function is relied on standards established by the Chinese Medical Association. The mean standard residual values of FEV1 are normally distributed and independent of height, age and gender.

### Data analysis

We first tested whether SNPs were in Hardy–Weinberg equilibrium. Logistic regression analyses were then performed to test the association between each SNP with COPD case/control status, and linear regression analyses were performed to assess the relationships between SNPs and quantitative phenotypes, *i.e.* pulmonary function measurements (percent predicted (pp) FEV1, ppFVC, and ppTLCO), inflammatory cells (eosinophils, lymphocyte, macrophage, neutrophil and sputum) and cytokines in sputum (IL-8, IL-6, TNFA and VEGF). Both regression analyses were adjusted based on the age, sex and pack-years smoked. Three genetic models were used: dominate model (homozygotes and heterozygotes for the minor allele being compared as a group with homozygotes for the major allele), codominant model (three genotype groups per SNP separately) and recessive model (homozygotes and heterozygotes for the major allele being compared as a group with homozygotes for the minor allele). Statistical analysis was performed using the PLINK software (version 1.07, http://pngu.mgh.harvard.edu/purcell/plink/) [32], and haplotype analysis was also conducted using the same software. P < 0.05 was considered statistically significant.

## Results

All genotype frequencies were consistent with Hardy-Weinberg equilibrium (p > 0.01). T1, T2 and Q-1 were significantly associated with COPD (p < 0.00004) adjusted for sex, age and pack-years smoked. In addition to controlling the p-value by the Benjamini-Hochberg method, we performed an alternative adjustment for multiple comparisons using a permutation-based approach. After this adjustment, these three SNPs still show significant associations with COPD (adjusted p < 0.00005).

Results of multiple regression analyses assuming a dominant model and codominant model are shown in the Additional file 1. Multiple regression analyses assuming a recessive model are presented below.

### Association of ADAM33 SNPs with inflammatory cells in sputum

**Figure 1:**
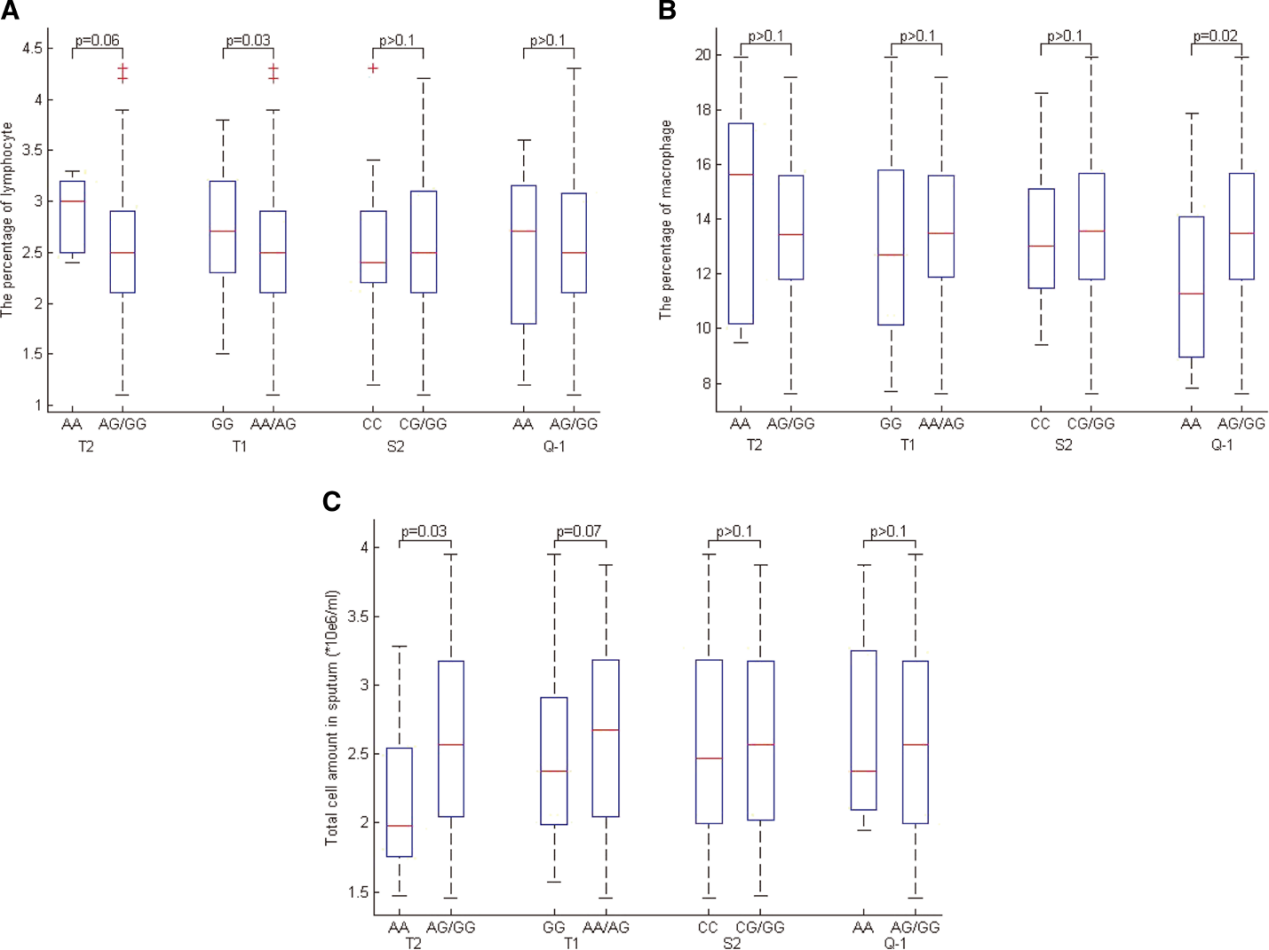
**Association of ADAM33 SNPs with the inflammatory cells in sputum under a recessive genetic model. A**: the percentage of lymphocyte; **B**: the percentage of macrophage; **C**: the total cell amount in sputum).

The analysis was conducted among cases only. The results showed that T1 was significantly associated with the percentage of lymphocyte (p = 0.03), Q-1 was associated with the percentage of macrophage (p = 0.02), and T2 was associated with total cell count in sputum (p = 0.03) under a recessive genetic model. T2 and T1 showed a trend toward association for the percentage of lymphocyte (p = 0.06) and total cell count in sputum (p = 0.07), respectively (Figure 1). However, no other significant association between these SNPs and other type of inflammatory cells was observed.

### Association of ADAM33 SNPs with cytokines in sputum

**Figure 2:**
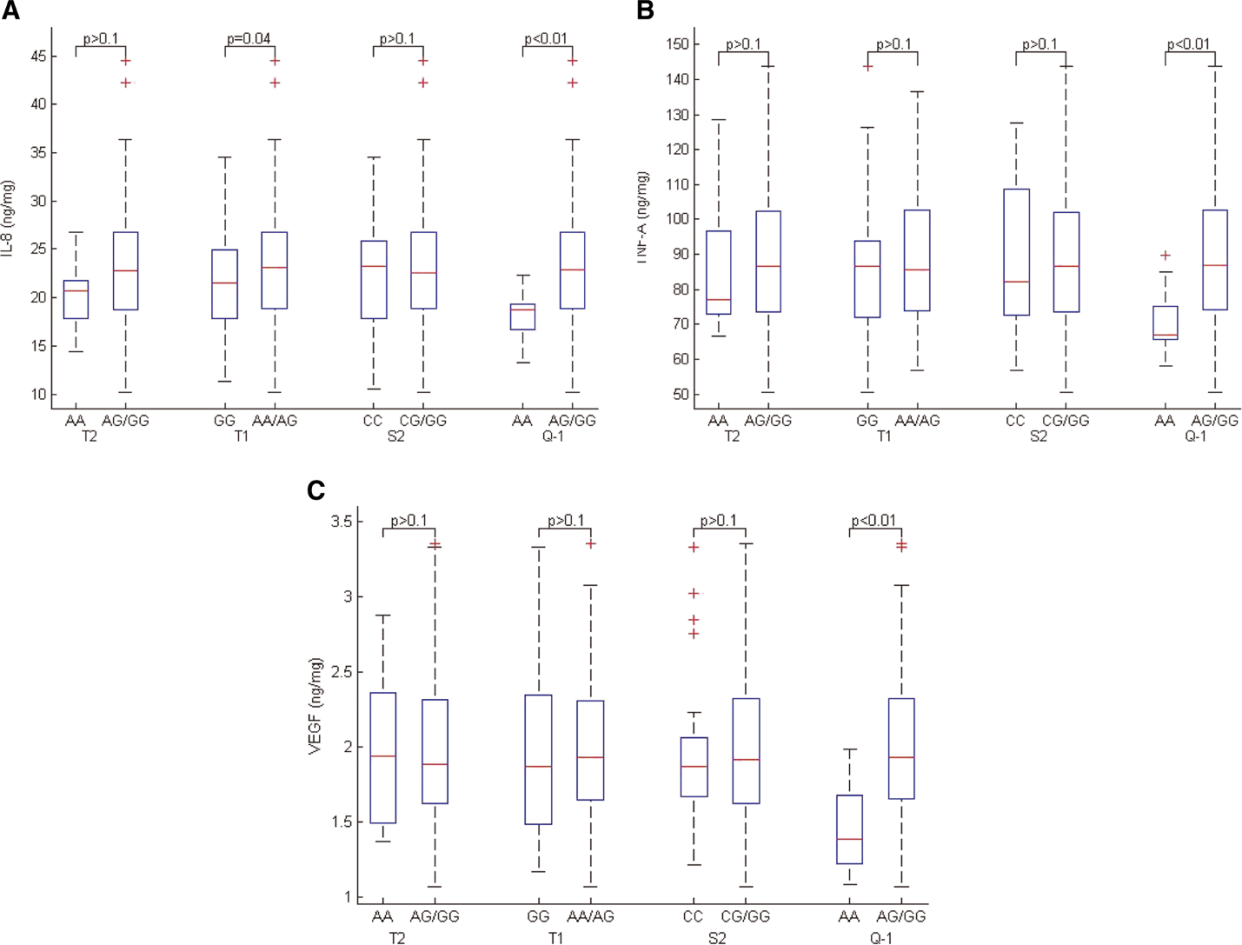
**Association of ADAM33 SNPs with cytokines in sputum under a recessive genetic model. A**: IL-8; **B**: TNF-A; **C**: VEGF.

There were no significant associations between IL-6 and these SNPs in recessive models. In contrast, the T1 SNP showed a significant association with IL-8 (p < 0.01) in subjects with COPD. The Q-1 SNP was associated with IL-8, TNF-A and VEGF (p < 0.01) (Figure 2). T2 and S2 were not associated with cytokines in sputum.

### Association ofADAM33 SNPs with pulmonary function

**Figure 3:**
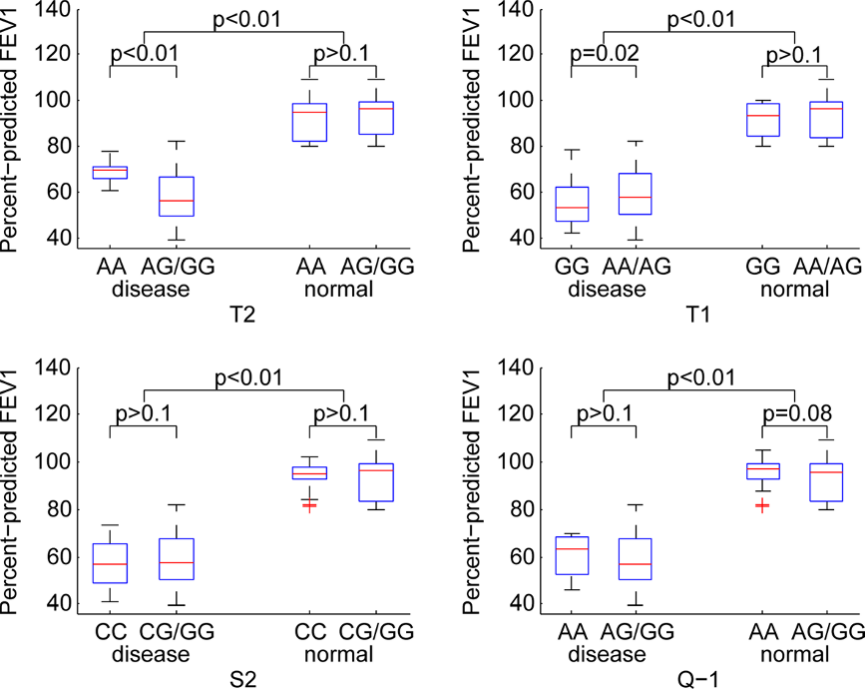
**Association of ADAM33 SNPs with ppFEV1 under a recessive genetic model.**

**Figure 4:**
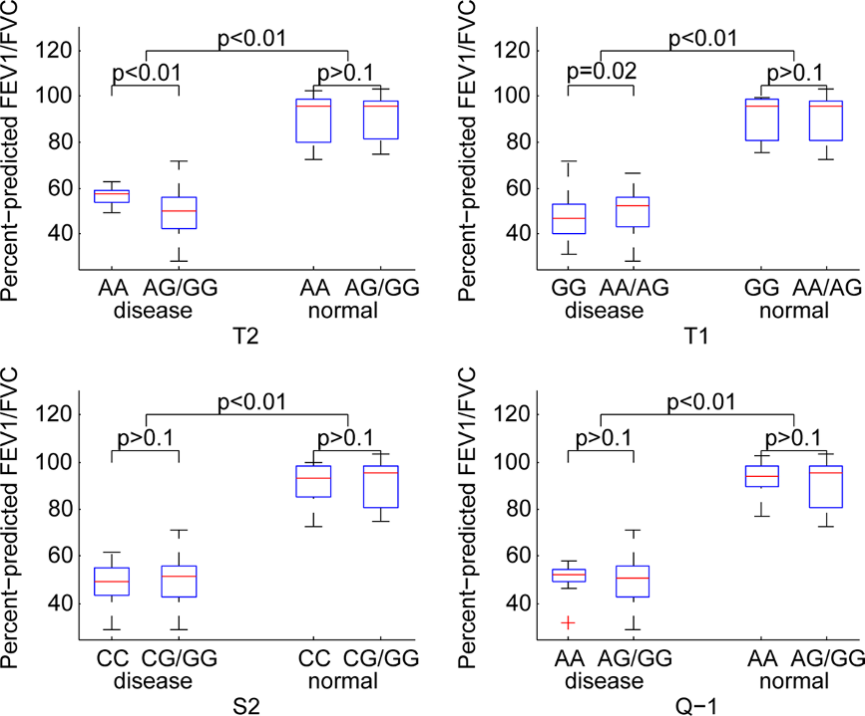
**Association of ADAM33 SNPs with ppFEV1/FVC under a recessive genetic model.**

**Figure 5:**
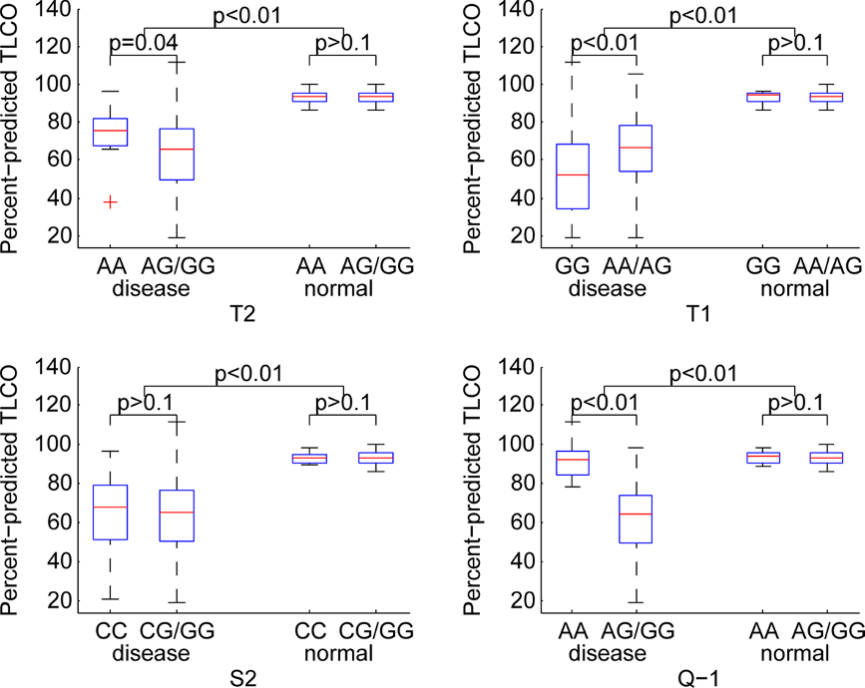
**Association of ADAM33 SNPs with ppTLCO under a recessive genetic model.**

The significant pulmonary functions were identified in the patients with any SNPs in comparison to the control samples. T2 and T1 were significantly associated with ppFEV1 (Figure 3), ppFEV1/FVC (Figure 4) and ppTLCO (Figure 5) within COPD cases in recessive models (p < 0.05). The Q-1 SNP was also significantly associated with ppTLCO (p < 0.01). When examining control samples only, there was no significant association between these SNPs and any of the measures of lung function.

### Haplotype association analysis

The haplotype associations with pulmonary function or inflammatory processes were investigated. 8 haplotypes on T2, T1, S2 and Q-1 were significantly different between cases and controls in either the crude analysis or the analysis adjusting for age, gender and smoking. Other 3 haplotypes were significantly different in the analysis adjusting for age, gender and smoking (Table 3).

**Table 3:** **Association of haplotypes with COPD in both the crude and the adjusted analysis**

				P-value	
T2	T1	S2	Q-1	Unadjusted	Adjusted ^*^
G	G	G	G	3.734e-011	1.1e-013
G	G	C	G	6.32e-009	1.72e-011
A	A	G	A	3.514e-006	3.76e-007
A	A	G	G	5.16e-006	8.81e-007
G	A	G	A	0.003103	0.0016
A	A	C	A	0.004037	0.000244
A	G	G	A	0.04011	0.00803
G	A	C	A	0.01269	0.00398
G	G	C	A	–	0.00712
G	G	G	A	–	0.0131
G	A	C	G	–	0.026
G	A	G	G	–	
A	A	C	G	–	
A	G	G	G	–	
A	G	C	G	–	

^*^The analysis adjusted for age, gender and smoking.

We investigated associations of haplotypes with cell count and cytokines in sputum with patients of COPD. GAGG, AGGG, AACG, AGCG and GGCA were found to be significantly associated with total cell count in sputum, GAGG was significantly associated with the percentage of lymphocyte, GACG was significantly associated with TNF-A, GACA, AAGA and GAGA were significantly associated with VEGF, and GAGG, AACG and AGCG was significantly associated with IL-8 in the analysis adjusting for age, gender and smoking (Table 4).

**Table 4:** **Association of haplotypes with inflammatory cells and cytokines in sputum in the adjusted analysis**

				P-value								
T2	T1	S2	Q-1	Sputum	Neutrophil	Macrophage	Lymphocyte	Eosinophils	TNF-A	VEGF	IL-6	IL-8
G	A	G	G	0.00207			0.0239					7.07e-006
A	A	C	G	0.00598								0.0322
A	G	G	G	0.00823								
A	G	C	G	0.0146								0.0435
G	G	C	A	0.0349								
G	A	C	G						0.0338			
G	A	C	A							0.00419		
A	A	G	A							0.0188		
G	A	G	A							0.0361		

We investigated associations of haplotypes with pulmonary function in COPD and controls in the analysis adjusted for age, gender and smoking. Only GAGA was significantly associated with ppFEV1 in the normal state. 10 haplotypes were significantly associated with ppTLCO in the disease state, 4 haplotypes (AAGG, GGCA, GGGA and AACG) were significantly associated with ppFEV1 and ppFEV1/FVC, while only GGGG was significantly associated with ppFEV1. AAGG, GGCA and GGGA were significantly associated with pulmonary functions (FEV1, FEV1/FVC and TLCO) (Table 5).

**Table 5:** **Association of haplotypes with pulmonary function in the analysis adjusted for age, gender and smoking**

				P-value					
T2	T1	S2	Q-1	Percent-predicted FEV1	FEV1/FVC	Percent-predicted TLCO	Percent-predicted FEV1	FEV1/FVC	Percent-predicted TLCO
				Disease			Normal		
G	G	G	G	0.0247	–	1.04e-005	–	–	–
G	G	C	G	–	–	0.000205	–	–	–
A	A	G	A	–	–	0.000368	–	–	–
A	A	G	G	0.00176	0.000154	1.67e-007	–	–	–
A	A	C	A	–	–	0.000224	–	–	–
G	A	G	A	–	–	–	0.005447	–	–
G	G	C	A	0.000234	0.000272	1.22e-005	–	–	–
A	G	G	A	–	–	0.0392	–	–	–
G	G	G	A	8.68e-005	0.00209	6.98e-016	–	–	–
G	A	G	G	–	–	0.00319	–	–	–
A	A	C	G	0.0154	0.0465	0.0014	–	–	–

9 different haplotypes (except GACA and GACG) were significantly associated with TLCO in either the crude analysis or the analysis adjusting for age, gender and smoking.

## Discussion

To our knowledge, this is the first study to show significant association between four polymorphisms (T1, T2, S2 and Q-1) of ADAM33 as well as their haplotypes and pulmonary function and airway inflammation of patients with COPD in an East Asian population. We first found that T1, T2 and Q-1 were significantly associated with COPD, while Figarsk et al. revealed that S1, S2, T2 and Q-1 were significantly associated with COPD in Vlagtwedde/Vlaardingen cohort [25]. Since no association was found between S1 and COPD in our previous study in a northeastern Chinese population [29], we did not include S1 in this study. We also found that T1, T2 and Q-1 were significantly associated with pulmonary function and components of cells in sputum of COPD, and T1 and Q-1 were significantly associated with cytokines and mediators of inflammation in airway of COPD in recessive models. These results suggest ADAM33 gene involved in the pathogenesis of COPD and multiple SNPs loci of interaction could promote the progress of COPD.

ADAM33 is a member of the zinc-dependent metalloproteinase ADAM superfamily, which regulate their own function and a wide range of other proteins through proteolytic cleavage [33]. ADAM33 has multiple domains including prodomain, catalytic, metalloprotease, disintegrin (binds integrins), cysteine-rich/epidermal growth factor (cell-cell contact), transmembrane, and cytoplasmic domains, and has multiple splice forms containing various combinations of these domains. ADAM33 is predominantly expressed in airway structural cells, including airway epithelium, airway smooth muscle (ASM), myofibroblasts, and fibroblasts, and is thought to have a wide spectrum of functions, such as protease-dependent and independent mechanisms. ADAMs cleaves proteins from the cell surface and might facilitate release of cytokines and growth factors. The pathogenesis of COPD involves the recruitment and regulation of neutrophils, macrophages, and lymphocytes to the lung, as well as the induction of imbalance between proteinases and antiproteinases, all of which result in lung parenchymal destruction and airway remodeling. Based on the expression profile and functions, ADAM33 is involved in pathogenesis of COPD. Our study showed that the SNP T1 is significantly associated with macrophage and T2 is significantly associated with total cells in sputum of patients with COPD, Q-1 was significantly associated with IL-8, TNF-A and VEGF in sputum of COPD. Therefore, like other ADAMs, ADAM33 can promote the release of cytokines and growth factors, lead to inflammatory cells infiltration in the airway. In addition, soluble ADAM33 has been implicated in angiogenesis [22] and new vessels, and may promote inflammatory cells and cytokines or growth factors in the airway. Although not as well studied, there is evidence that the vascular areas of the airway is significantly increased in COPD and are related to the degree of airflow obstruction [34]. In this current study, our results showed that T1 and Q-1 affected lymphocyte and macrophage, implying that ADAM33 might affect the immune response of the airways and further promote the formation of COPD. Some studies have also found the association between macrophage and lymphocytes and immune responses of COPD. For example, Winkler et al. found that the products of activated macrophages have also been implicated in inflammation and tissue destruction, including in COPD [35]. Domagala-Kulawik et al. showed in their study that macrophages were involved in the inflammatory process caused by smoking in COPD and were associated with severe airflow limitation [36].

In the airway of patients with COPD, the inflammation limits the airflow and further causes a loss of lung function of COPD. It is known that pulmonary function decline is a prominent characteristic for the development of COPD and cardiovascular disease [37]. Our data confirmed that T1 and T2 were significantly associated with decreased FEV1 and FEV1/FVC, T2, T1 and Q-1 were significantly associated with decreased TLCO. The study conducted by Jongepier H and colleagues found similar results in asthmatic patients [38]. It is important to note that ADAM33 SNPs have also shown associations with excess FEV1 decline in general population samples. The main SNPs identified included Q-1, S1, S2 with excess decline of 23.6 to 9.6 mL/y. S1 and S2 were associated with COPD in this population, reflecting the potential role of ADAM33 in airway remodeling in multiple respiratory diseases. Emphysema is another main pathological condition for COPD [39]. It’s worth noting that, in comparison to other studies, our study also identified the relationship between SNP and TLCO of COPD since TLCO associated with the severity of emphysema. We found that T2, T1 and Q-1 were associated with decreased TLCO of COPD, suggesting that the ADAM33 also play a role in the pathogenesis of emphysema. An imbalance of endogenous proteinases and antiproteinases is considered to be a major mechanism for emphysematous lung destruction. In this context, matrix metalloproteinase have aroused interest due to their capability of inducing emphysema by proteolysis of lung parenchyma. Similar to matrix metalloproteinase, the ADAM family possesses proteolytic and adhesive activity. As a member of this gene family, any alterations of ADAM33 may affect the regulation of cell-cell and cell-matrix adhesion and the extent of extracellular matrix degradation. Therefore, it is conceivable that mutations in ADAM33 might promote emphysematous changes, a main phenotype or pathologic change of COPD.

We found that T1, T2 and Q-1 were significantly associated with COPD. The SNP Q-1 is located in the intron immediately before exon 16, which contains an epidermal growth factor (EGF) domain [40]. EGFR signaling regulates matrix metalloprotease, which mediates epithelial-mesenchymal interactions during lung morphogenesis [41]. The mice lacking the EGF receptor (EGFR) demonstrate abnormal branching and poor alveolarization. ADAM33 is closely related to matrix metalloprotease, but may bind EGF directly. Through alterative splicing, exon 16 can be spliced out, giving rise to the β -variant of ADAM33. This variant was found in 30% of ADAM33 mRNA transcripts in pulmonary fibroblasts [42]. The intronic Q-1 SNP has been identified to influence the splicing of the β–variant [43], and disturb the maturation of ADAM33. Because the EGF domain is incomplete, it has been suggested that the β –variant prevents maturation of ADAM33 and may exert a dominant-negative effect on its protease activity [44]. Subsequent effects on protease activity may result in a defect in tissue repair after inflammation-induced damage. This may lead to progressive destruction of alveolar tissue and thereby enhance accelerated decline in lung function of COPD.

The T1 and T2 SNPs are located in the exon 19 (which includes an SH3 domain and a phosphorylation site) of the cytoplasmic tail, which may affect signaling [29]. T1, T2 are in the domain Reprolysin family propeptide, which interacts with 5 domains, including Thrombospondin, C-type lection and ADAM-TS Spacer 1. Thrombospondin 1 is an antiangiogenic factor, which can inhibit the proliferation and migration of endothelial cells. Angiogenesis is a key process in the evolution and maintenance of psoriasis and other chronic inflammatory diseases, such as inflammatory bowel disease, chronic obstructive pulmonary disease, and rheumatoid arthritis [45]. Proteins that contain C-type lectin domains have a diverse range of functions including cell-cell adhesion, immune response to pathogens and apoptosis. Apoptosis has been recognized to play an important role in clinical and experimental models of lung diseases [46]. Recently many reports have been published highlighting the presence of apoptosis in human COPD lungs and its role in the pathogenesis of emphysema [47]. ADAM-TS Spacer1 represents the Spacer-1 region from the ADAM-TS family of metalloproteinase and ADAM-TS family has some lung-related biological actions [48].

In this current study, we also analyzed associations between haplotypes of four polymorphisms (T1, T2, S2 and Q-1) in ADAM33 and pulmonary function and airway inflammation of patients with COPD. GAGG, AGGG, AACG, AGCG and GGCA were found to be significantly associated with total cell count in sputum: GAGG was significantly associated with increase lymphocyte count; GACG was significantly associated with increased level of TNF-A; GACA, AAGA and GAGA were significantly associated with VEGF; and GAGG, AACG and AGCG was significantly associated with IL-8 in the analysis adjusted for age, gender and smoking. These results demonstrated that multiple SNPs could work together to promote airway inflammation of patients with COPD. It is worth noting that 10 haplotypes were found to be significantly associated with ppTLCO in the disease state, 5 of these haplotypes (AAGG, GGCA, GGGA, AACG and GGGG) were significantly associated with ppFEV1, and 4 haplotypes (AAGG, GGCA, GGGA and AACG) were significantly associated with ppFEV1/FVC. These results indicated that although there were no common associations between single SNP and inflammatory cells in sputum, haplotypes of these SNPs were found to be associated with the quantitative traits. COPD high-risk groups could be screened out using these haplotypes of ADAM33 in the future, though much effort should be put into this clinical field.

We focused on the association of four polymorphisms (T1, T2, S2 and Q-1) of ADAM33 as well as their haplotypes with pulmonary function and airway inflammation of patients with COPD, thus, no sputum cells were obtained from control samples. More comprehensive data should be obtained to reveal the mechanism of COPD in further study.

## Conclusions

In this paper, we confirmed for the first time that ADAM33 was involved in the pathogenesis of COPD in an East Asian population by affecting airway inflammation and immune response. Our results made the genetic background of COPD, a common and disabling disease, more apparent, which would supply genetic support for the study of mechanism, classification and treatment for this disease.

## Electronic supplementary material

Additional file 1: **Results of multiple regression analyses assuming a dominant model and codominant model.** (DOC 1 MB)
