# Supplementary material for: Genetic variants in ADAM33 are associated with airway inflammation and lung function in COPD
Source: BMC Pulm Med. 2014 Nov 4;14:173. doi: 10.1186/1471-2466-14-173 (PMC4228268; doi:10.1186/1471-2466-14-173)
Supplement: Supplementary file 1 — Additional file 1: Results of multiple regression analyses assuming a dominant model and codominant model. (DOC 1 MB) [file 12890_2013_607_MOESM1_ESM.doc]

**Dominant genetic model**

**Association of ADAM33 SNPs with inflammatory cells in sputum**

The analysis was conducted among cases only. The results showed that T2 and S2 was associated with total cell count in sputum (p<0.05) under a dominant genetic model. T1 showed a trend toward association for the percentage of macrophage (p=0.05) (Figure S1). However, no other significant association between these SNPs and other type of inflammatory cells was observed.


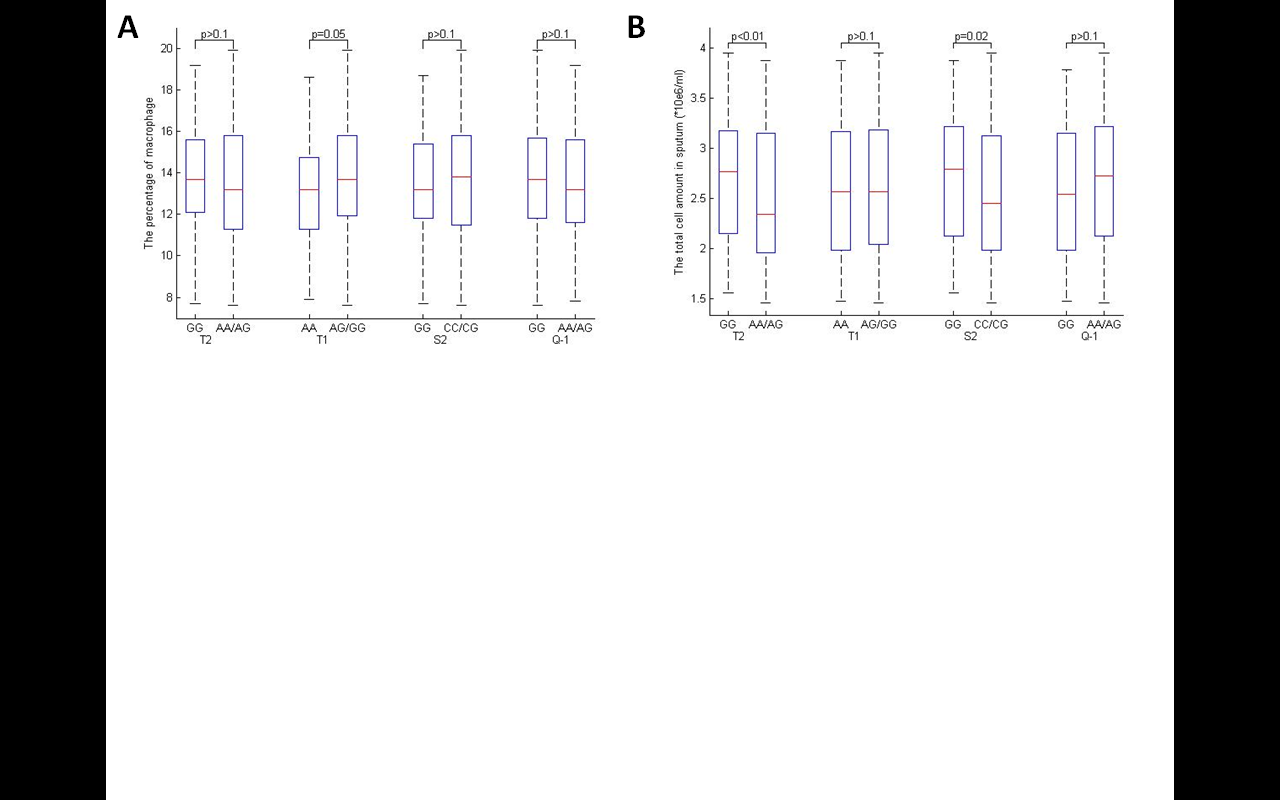


**Figure S1. Association of ADAM33 SNPs with inflammatory cells (A: the percentage of macrophage; B: the total cell amount in sputum) in sputum** **under a dominant genetic model.**

**Association of ADAM33 SNPs with** [**cytokines**](app:ds:cytokines) **in sputum**

There were no significant associations between IL-6 and these SNPs in dominant models. In contrast, the S2 and Q-1 SNPs showed a significant association with IL-8 (p<0.05) in subjects with COPD. The T2 SNP were associated with TNF-A (p=0.03) and showed a trend toward association for VEGF (p=0.05). Q-1 were associated with VEGF (p<0.03) and showed a trend toward association for TNF-A (p=0.05). T1 showed a trend toward association for IL-8 (p=0.06) (Figure S1). However, no other significant association between these SNPs and other type of cytokines was observed.


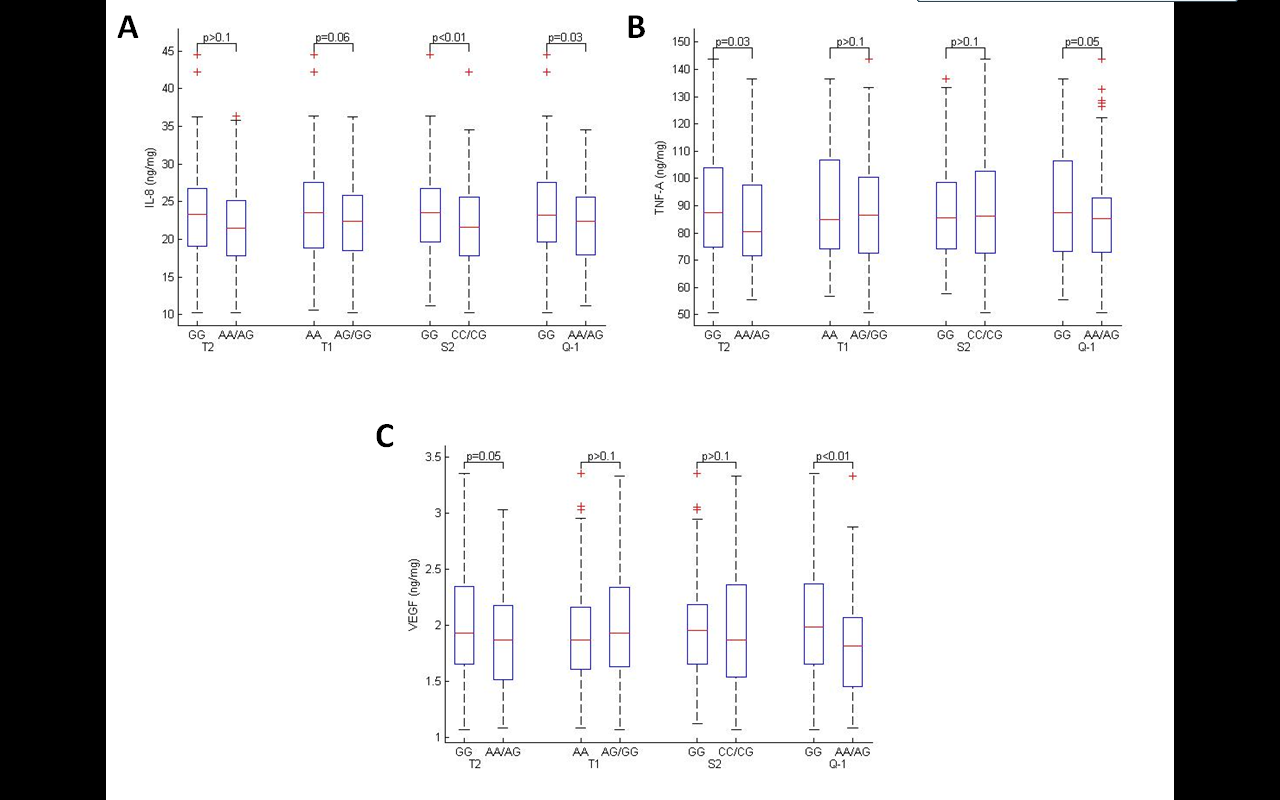


**Figure S2. Association of ADAM33 SNPs with cytokines (A: IL-8; B: TNF-A; C: VEGF) in sputum under a dominant genetic model.**

**Association ofADAM33 SNPs with pulmonary function**

Pulmonary functions for all SNPs were significantly different between cases and controls.

T2, T1, and Q-1 were significantly associated with ppFEV1 (Figure S3), ppFEV1/FVC (Figure S4) and ppTLCO (Figure S5) within COPD cases in dominant models (p<0.05). When examining control subjects only, there was no significant association between these SNPs and any of the measures of lung function.


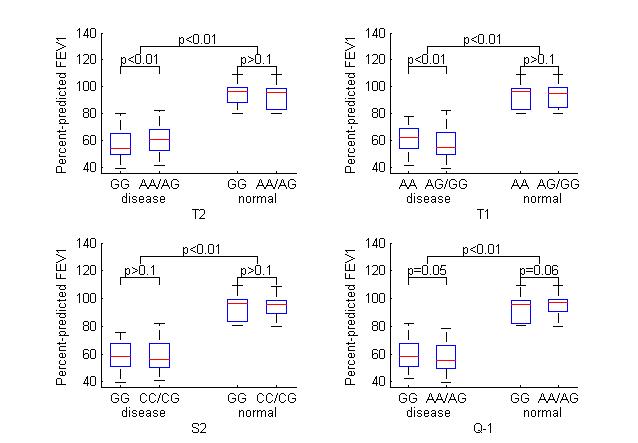


**Figure S3. Association of ADAM33 SNPs with ppFEV1 under a dominant genetic model.**


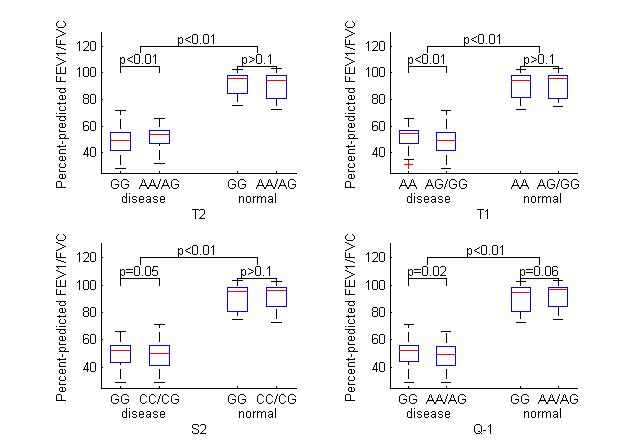


**Figure S4. Association of ADAM33 SNPs with ppFEV1/FVC under a dominant genetic model.**


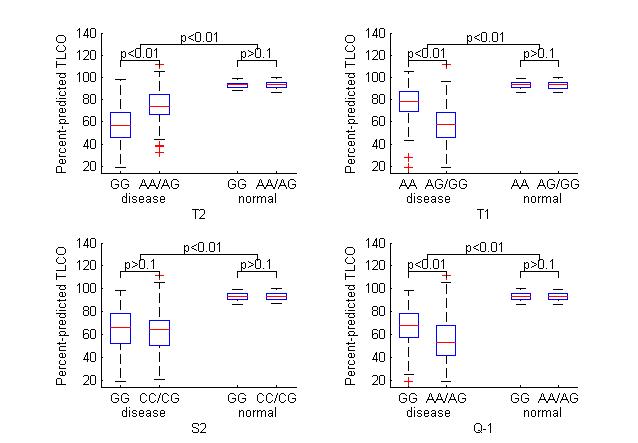


**Figure S5. Association of ADAM33 SNPs with ppTLCO under a dominant genetic model.**

**Codominant genetic model**

**Association of ADAM33 SNPs with inflammatory cells in sputum**

The analysis was conducted among cases only. The results showed that T1 was significantly associated with the percentage of macrophage (p=0.03), and T2 was associated with total cell count in sputum (p<0.01) under a codominant genetic model. Q-1 and S2 showed a trend toward association for the percentage of lymphocyte (p=0.06) and total cell count in sputum (p=0.06), respectively (Figure S6). However, no other significant association between these SNPs and other type of inflammatory cells was observed.


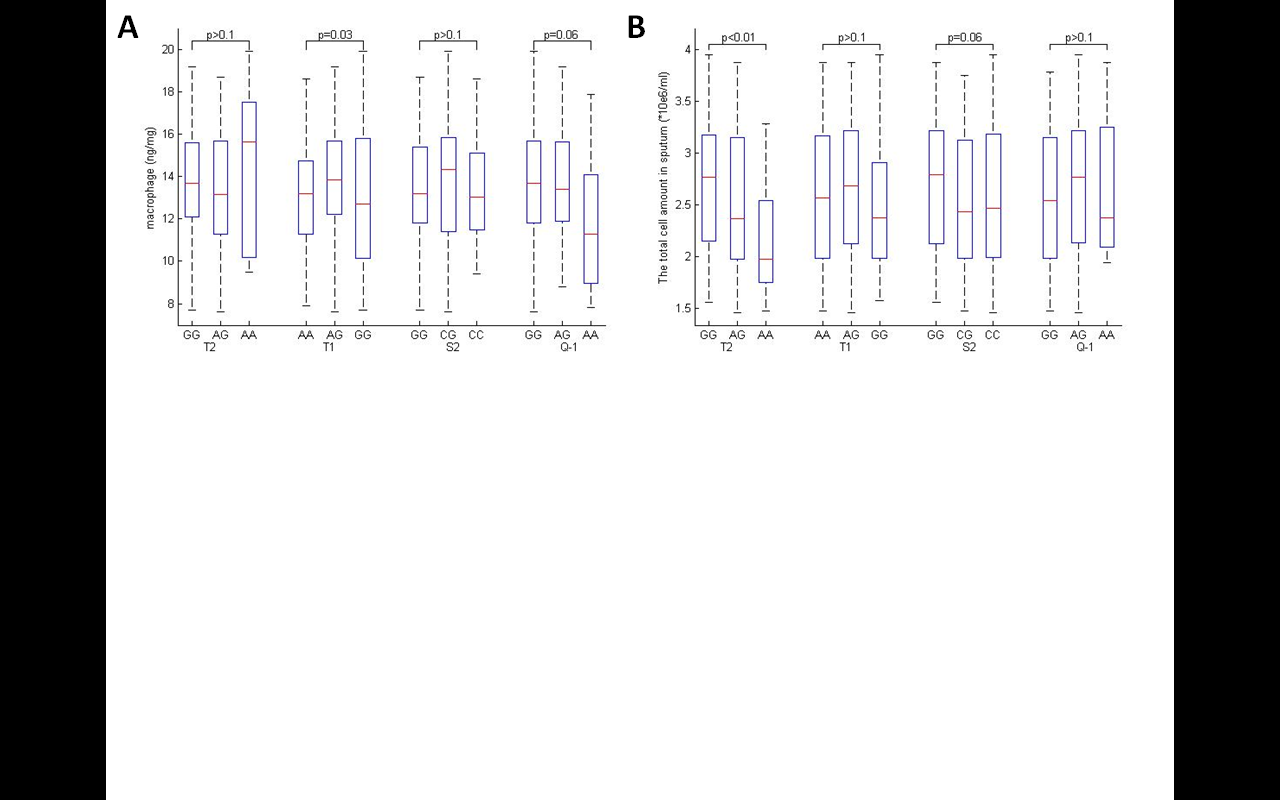


**Figure S6. Association of ADAM33 SNPs with inflammatory cells (A: the percentage of macrophage; B: the total cell amount in sputum) in sputum** **under a codominant genetic model.**

**Association of ADAM33 SNPs with** [**cytokines**](app:ds:cytokines) **in sputum**

There were no significant associations between IL-6 and these SNPs in codominant models. In contrast, the S2 SNP showed a significant association with IL-8 (p<0.01) in subjects with COPD. The Q-1 SNP were associated with IL-8, TNF-A and VEGF (p<0.01) (Figure S7). T2 and T1 were not associated with cytokines in sputum.


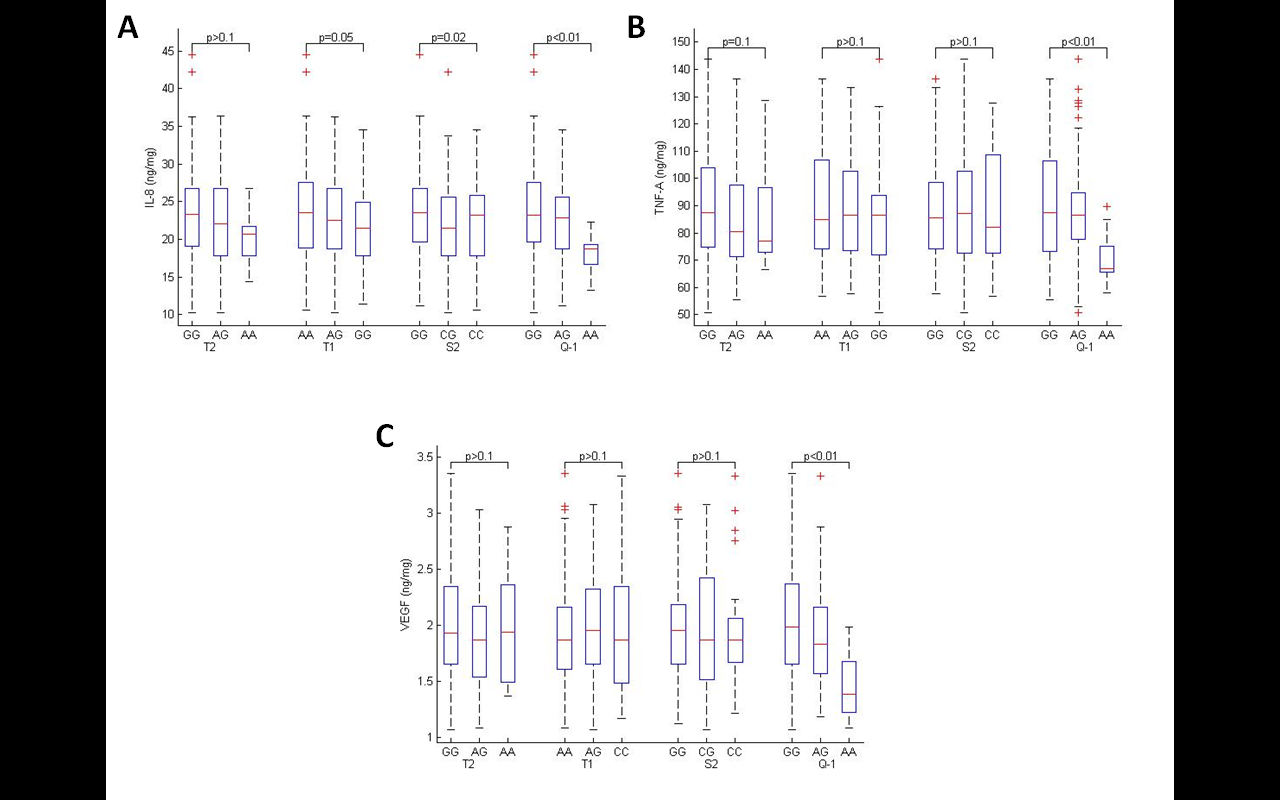


**Figure S7. Association of ADAM33 SNPs with cytokines (A: IL-8; B: TNF-A; C: VEGF) in sputum under a codominant genetic model.**

**Association ofADAM33 SNPs with pulmonary function**

Pulmonary functions for all SNPs were significantly different between cases and controls.

T2 and T1 were significantly associated with ppFEV1 (Figure S8), ppFEV1/FVC (Figure S9) and ppTLCO (Figure S10) within COPD cases in codominant models (p<0.05). The Q-1 SNP was also significantly associated with ppFEV1/FVC and ppTLCO (p<0.01). When examining control subjects only, there was no significant association between these SNPs and any of the measures of lung function.


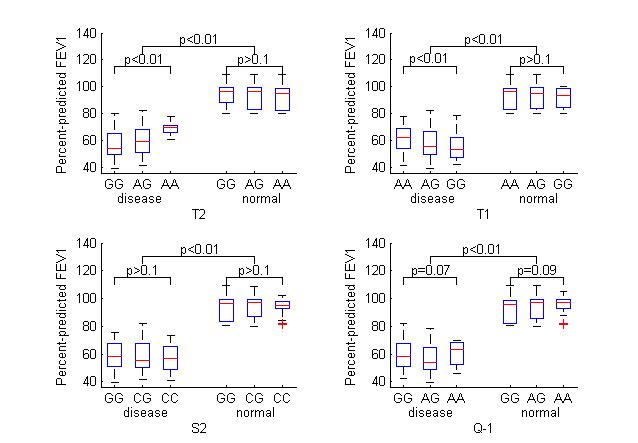


**Figure S8. Association of ADAM33 SNPs with ppFEV1 under a codominant genetic model.**


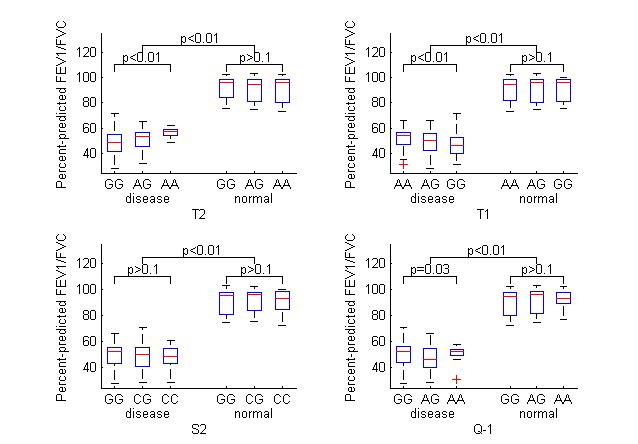


**Figure S9. Association of ADAM33 SNPs with ppFEV1/FVC under a codominant genetic model.**


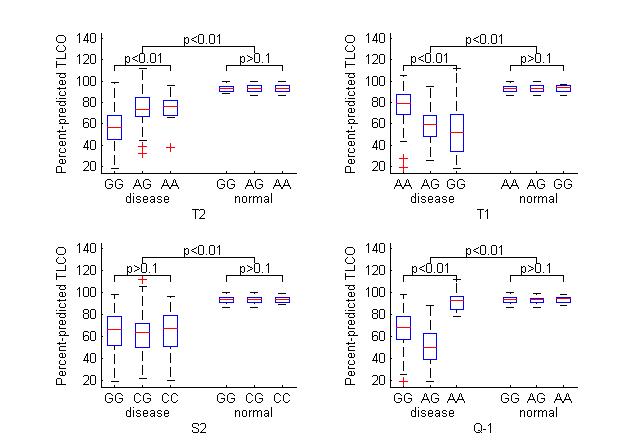


**Figure S10. Association of ADAM33 SNPs with ppTLCO under a codominant genetic model.**
